# A longitudinal study of the impact of chronic psychological stress on health-related quality of life and clinical biomarkers: protocol for the Australian Healthy Aging of Women Study

**DOI:** 10.1186/1471-2458-14-9

**Published:** 2014-01-08

**Authors:** Charrlotte Seib, Eliza Whiteside, Janice Humphreys, Kathryn Lee, Patrick Thomas, Lisa Chopin, Gabrielle Crisp, Angela O’Keeffe, Michael Kimlin, Andrew Stacey, Debra Anderson

**Affiliations:** 1 Institute of Health and Biomedical Innovation, Queensland University of Technology, Victoria Park Road, Kelvin Grove, QLD 4059, Australia; 2 School of Nursing, 2 Koret Way, University of California, San Francisco, San Francisco, CA 94143-0606, USA; 3 NHMRC Center for Research Excellence in Sun and Health, Queensland University of Technology, Victoria Park Road, Kelvin Grove, QLD 4059, Australia

**Keywords:** Women, Stress, Health-related quality of life, Stress biomarkers, Modifiable lifestyle factors, Midlife

## Abstract

**Background:**

Despite advancements in our understanding of the importance of stress reduction in achieving good health, we still only have limited insight into the impact of stress on cellular function. Recent studies have suggested that exposure to prolonged psychological stress may alter an individual’s physiological responses, and contribute to morbidity and mortality. This paper presents an overview of the study protocol we are using to examine the impact of life stressors on lifestyle factors, health-related quality of life and novel and established biomarkers of stress in midlife and older Australian women.

The primary aim of this study is to explore the links between chronic psychological stress on both subjective and objective health markers in midlife and older Australian women. The study examines the extent to which exposure frightening, upsetting or stressful events such as natural disasters, illness or death of a relative, miscarriage and relationship conflict is correlated with a variety of objective and subjective health markers.

**Methods/Design:**

This study is embedded within the longitudinal Healthy Aging of Women’s study which has collected data from midlife and older Australian women at 5 yearly intervals since 2001, and uses the Allostastic model of women’s health by Groër and colleagues in 2010. The current study expands the focus of the HOW study and will assess the impact of life stressors on quality of life and clinical biomarkers in midlife and older Australian women to explain the impact of chronic psychological stress in women.

**Discussion:**

The proposed study hypothesizes that women are at increased risk of exposure to multiple or repeated stressors, some being unique to women, and the frequency and chronicity of stressors increases women’s risk of adverse health outcomes. This study aims to further our understanding of the relationships between stressful life experiences, perceived quality of life, stress biomarkers, chronic illness, and health status in women.

## Background

### The impact of chronic psychological stress

The potentially negative impact of social issues and stressful life events (SLEs) on health and wellbeing is increasingly recognised as a significant public health issue. Indeed, decrements in health status and quality of life have been attributed to social factors such as poverty, early life experiences, the presence or absence of social supports, employment status, the availability of food sources, transport options and characteristics of the work environment [1]. Other studies have suggested that witnessing or experiencing violence, particularly intimate partner violence [2,3], political violence [4-6] and child maltreatment [3,7], may increase one’s risk of developing depressive symptoms. Others have linked stressful events such as war and conflict, natural disasters, academic failure, injury, job loss, major financial crises, divorce, illness or death of a loved one to the experience of poor health [8-11].

Studies in women specifically have suggested that social health issues and stressful life events are related to a range of physical and emotional health problems, and reduced health-related quality of life (HRQoL). These include increased sexual and reproductive health problems, chronic pain, somatic conditions, gastrointestinal disorders, suicidal ideation and risk-taking behaviours [12]. Moreover, a recent Australian study suggested that, compared to women with no social health issues, women reporting three or more stressful life events or social health issues have a twofold increased risk of having a low birth weight baby [13].

Whether women are particularly susceptible to life stressors is unclear. Some studies of sex-specific responses to stress have found that women are more likely to develop depression than men following a traumatic event [14,15], while others have found no such association [16-18]. It may be that women report poor health because of an increased risk of multiple or repeated exposures to life stressors [13,17,18]. Indeed, the likelihood of major depression increased 10-fold among those reporting four severe stressful events (including the death of a close relative, assault, serious marital problems or relationship breakup) in one month [19].

The cumulative burden of chronic life stress may exceed the body’s ability to repair it’s physiological systems, leading to increased wear and tear, and dysregulation which may lead to pathophysiological responses [20,21]. Indeed, recent studies have suggested that life stressors may impact on more than just mental health and may in fact alter one’s physiology [9]. Current work has indicated that chronic stress associated with childhood adversity, intimate partner violence or being someone’s carer may increase one’s risk of illness and premature death with or without the continued presence of the stressor [22-25]. It has been theorized that this occurs because prolonged exposure to stress hormones alters many physiological systems, including metabolic and inflammatory pathways, and immunological defense systems in the body which can lead to impaired functions and cell senescence [22-25].

In this study, we aim to define the links between chronic psychological stress on the health and quality of life of Australian women through the use of a unique mixed methods approach which we describe below.

## Methods

### Aims and hypothesis

The primary aim of this study is to explore the links between chronic psychological stress on subjective and objective health markers in midlife and older Australian women. The study will examine the extent to which exposure to a range of frightening, upsetting or stressful events such as natural disasters, illness or death of a relative, miscarriage and relationship conflict is correlated with a variety of objective and subjective health markers. It is hypothesized that women are at increased risk of exposure to multiple or repeated stressors, some being unique to women, and the frequency and chronicity of stressors may increase women’s risk of poor health outcomes. This study will address three main research questions:

(1) What is the impact of stressful life events on health-related quality of life (HRQoL)?

(2) Do women who report multiple stressors report worse HRQoL than women who report one or no SLEs?

(3) Do women who report exposure to SLEs, and who have poor HRQoL, also show corresponding changes in clinical biomarkers of stress and cellular aging?

### Theoretical framework

Recent studies have suggested that chronic psychological stress may accelerate aging and increase susceptibility to many of the common risk factors associated with morbid health conditions [26]. Rather than being associated with a singular isolated stressful event however, it may be the many and varied daily stressors that predict physiological dysregulation [27]. Indeed, exposure to multiple or repeated stressors over time causes continual adjustment of neural, endocrine and immune stress mediators in order to maintain homeostasis, leading to maladaptation and a range of poor health outcomes [27,28]. This process is referred to allostatic load (AL) [28].

The allostatic load (AL) framework provides a basis for understanding the cumulative physiologic effects of adaption to stress throughout the lifespan [29]. The AL framework includes several components; primary mediators (neural, immune and metabolic biomarkers), secondary outcomes (cardiovascular, respiratory and anthropometric) and morbidity overtime [26,27,29]. Research suggests the higher the allostatic load, the more extensive the physiologic dysregulation, and a higher the risk of illness and premature death [21,27,29-32].

**Figure 1:**
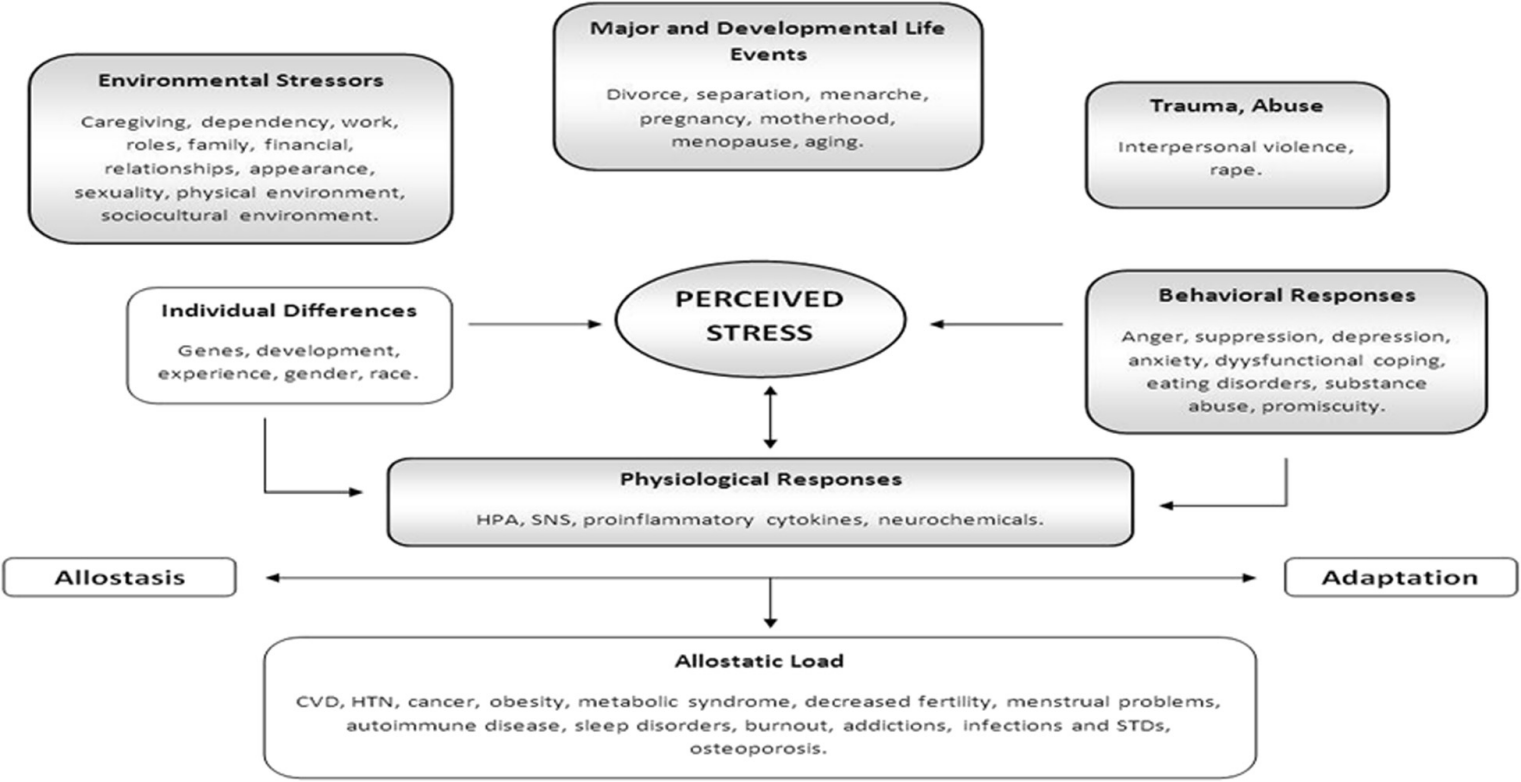
**An allostatic model of women’s health **[20]**, p.186.**

Of course, people respond to stress differently. Indeed, stress response is linked to the way in which an individual evaluates a perceived threat and also their ensuing psychological response, and variations may be associated with genetic, epigenetic, developmental, and experiential factors [27,30,33]. To help understand the potentially deleterious effects of stress on women’s health, the following model [20] has been used to guide this research. This study examines correlations between environmental stressors, major life events and trauma and abuse and HRQoL, depressive symptoms poor lifestyle behaviors (behavioral responses to stress), and physiological responses (measured as clinical biomarkers and diagnosis of diseases) (Figure 1).

### Participants

This study is embedded within the longitudinal Healthy Aging of Women’s (HOW) study which has collected data from midlife and older Australian women at 5 yearly intervals since 2001. The original sample was drawn from 10,923 eligible women on the electoral roll in 2000. Inclusion criteria were: (1) women aged 45-60 years; (2) currently residing in the six selected rural and metropolitan postcodes within Queensland, Australia. From that target population, 1500 women were randomly selected for Stage 1 of the study in 2001. Of these, 869 women (59% response) participated.

The second survey, five years later in 2006, was mailed to 866 participants, since two had indicated that they no longer wished to participate, and notification was received that another was deceased. Completed surveys were received from 564 women aged 51-66 years. A third time wave has been conducted in March in 2011. Surveys were sent to remaining participants (68% response rate) and data were received from 350 women aged 60 to 70 years.

Data comparing the retained sample with those who were lost to follow-up revealed that women who were lost to follow-up were more likely to be divorced or separated (χ2 (3) = 8.475, p = 0.037), unable to work due to prolonged illness (χ2 (3) = 12.389, p = 0.030), and were less likely to engage in screening activities (pap smears, χ2 (1) = 4.561, p = 0.033; mammograms, χ2 (1) = 13.898, p <0.001 respectively). Further analysis however showed that women did not differ across most key variables socio-demographic variables (country of birth, p = 0.156; educational attainment, p = 0.271; income, p = 0124), health variables (PCS, p = 0.146; MCS, p = 0.990), or modifiable lifestyle factors (BMI, p = 0,962; cigarette smoking, p = 0.235, alcohol consumption, p 0.605; exercise, p = 0.095). Finally, differences in age, though statistically significant, were small and not clinically important (mean difference -0.4715, p = 0.008).

Data amassed from the HOW study over the last decade (2001, 2006, 2011) suggests that modifiable lifestyle factors are predictive of health status in this sample [34-45]. Further preliminary bivariate analysis of 2011 data suggests that, while quality of life is not associated with alcohol or caffeine consumption, it is negatively associated with difficulty sleeping (PCS: r = -0.210, p <0.001; MCS: r = -0.161, p = 0.006) and positively associated with physical activity (PCS: F = 10.843, df = 4,271, p <0.001; MCS: F = 2.648, df = 4,271, p = 0.034). However, one limitation of the HOW study is a lack of available information about life stressors, which are increasingly recognized as predictors of illness and premature death [22-25,46].

The current study expands the current focus of the HOW study and will assess the impact of life stressors on HRQoL and clinical biomarkers in midlife and older Australian women. This component of the research will collect three types of data: 1) type and chronicity of SLEs from a structured questionnaire; 2) buccal cells from a self-administered buccal cell swab, and; 3) fasting blood from samples obtained by a pathology collection centre of their choice.

This study has been approved by Queensland University of Technology Human Research Ethics Committee (Ethical approval number 1100000171). All participants will be asked to provide informed written consent prior to commencing the study.

### Measures

#### ***Stress questionnaire***

Participating women were asked to complete an additional brief questionnaire about experiences of stress and conflict. The questionnaire included two measures; the Life Stressor Checklist – Revised (LSC-R) [11] and the Revised Conflict Tactics Scale (CTS2) [47].

The LSC-R is a 30 item questionnaire used to assess events in a person’s life that women may have found frightening or upsetting like natural disasters, sexual or physical assault and illness or death of a relative [11] and also includes items more relevant to women like miscarriage and abortion, or being unwillingly separated from children. Participants were asked about exposure to stressful event, their age at the time of exposure, the duration of the event, and the extent to which the event has affected them in the past 12 months. The LSC-R is designed to screen for life events that meet DSM-IV criteria for trauma but also includes items related to participants feelings and general distress related to the event [48]. The instrument has been used in diverse groups of women and had demonstrated adequate test–retest reliability and good criterion-related validity [11,49,50]. In this study, the number of lifetime trauma exposures is calculated by summing endorsed items, with higher scores representing exposure to more stressful life events.

The CTS2 is one of the most widely used instruments to assess aggression and conflict within intimate relationships [47,51-54]. The instrument consists of 78 items assessing both perpetration of violence and also victimization; the present study uses only the 39 items relating specifically to women’s victimization experiences [53]. Items are summed and converted into five conflict resolution subscales: psychological aggression, sexual coercion, negation, physical assaults, and injury experiences [23]. These scales examine actions taken to settle a disagreement (negotiation); acts of verbal and non-verbal aggression (psychological aggression); physical violence (physical assault); unwanted sexual activity (sexual coercion), and; physical pain or injury requiring medical attention (injury) [51].

#### ***Potential behavior responses to stress***

Women were also asked to complete a range of questions about health and lifestyle behaviors that might be impacted by exposure to stressful life experiences. The instruments included in this study were: (1) the Medical Outcomes Study Short Form 12 (SF-12®), a widely used measure of health-related quality of life [55]; (2) the Center for Epidemiologic Studies Depression Scale (CES-D), a 20-item instrument that examines recent depressed mood or affect [56], and; a several items related to modifiable lifestyle behaviors like body mass index [57], physical activity [36,58], alcohol and tobacco use [59], fruit and vegetable consumption [60], and sleep disturbance [61].

#### ***Buccal cell DNA collection***

Women were asked to provide a specimen of buccal cells using a collection protocol previously outlined by Richards and colleagues [62]. Briefly, the participant washed her mouth with water twice, then using gloved hands and a sterile buccal cytobrush (Gentra Puregene Buccal Cell Kit, QIAGEN, Germantown, MD), gently scraped the inside of her cheek ten times. The brush was then transferred into a sterile tube for transportation back to the laboratory at room temperature. Genomic DNA was extracted from buccal swabs within 48 hours of collection, following the manufacturer’s instructions (Gentra Puregene Buccal Cell Kit, QIAGEN). DNA integrity and concentration in each sample were analyzed using a Nanodrop 1000 spectrophotometer (Thermo Fisher, Scoresby, VIC, Australia). DNA was stored at -80°C until analyses were performed.

#### ***Serology***

Participants were also asked to contribute a fasting morning blood sample through their local pathology service centre. Two vials of blood were collected in EDTA tubes and transported to our QUT laboratory where blood samples were separated by centrifugation (3300× *g* for 10 minutes) to separate the buffy coat, plasma and red blood cells. Three aliquots of each fraction were collected and stored at -80°C until analyses were performed.

#### ***Cortisol levels***

Morning, fasting cortisol levels were measured in participant’s plasma samples using a Liaison Cortisol kit (Reference 313261, DiaSorin, Italy).

#### ***Buffy coat DNA collection***

Genomic DNA was extracted from buffy coat specimens using a QIAamp Blood and Body Fluid Spin Kit (QIAGEN) and DNA integrity and concentration in each sample was analyzed using a Nanodrop 1000 spectrophotometer (Thermo Fisher, Scoresby, VIC, Australia). DNA was stored at -80°C until analyses were performed.

#### ***Relative telomere length assay by quantitative polymerase chain reaction (PCR)***

This method was based on the protocol described by Cawthon [63]. Two master mixes of PCR reagents were prepared using SYBR Green PCR Master Mix (Applied Biosystems, Foster City, CA), one with telomere primers and one with primers for hbg (encoding the haemoglobin gamma subunit, a single copy gene). Telomere master mix (10 μl) was added to each well of the first plate and 10 μl hbg master mix was added to the second plate. MicroAmp Fast Optical 96-well reaction plates were used (Applied Biosystems, Foster City, CA). For each standard curve, a reference DNA sample from the MDA-MB-231 breast cancer cell line were serially diluted fivefold in ultrapure distilled water to produce five concentrations ranging from 0.064 ng/μl to 40 ng/μl. Dilutions were then distributed in 2.5 μl aliquots to the standard curve wells on each plate. For each DNA sample (from buccal cells or buffy coat cells), four identical 2.5 μl aliquots were added to plate one and another four added to plate two. The plates were sealed with a MicroAmp Clear Adhesive Film (Applied Biosystems) and centrifuged briefly. Final primer concentrations were 200 nM. Primer sequences (5’to 3’) were: tel2b, GGCTTGCCTTACCCTTACCCTTACCCTTACCCTTACCCT; tel1b, CGGTTTGTT TGGGTTTGGGTTTGGGTTTGGGTTTGGGTT; hbg2, CACCAACTTCATCCACGTTCACC; hbg1, GCTTCTGACACAACTGTGTTCACTAGC.

All PCRs were performed on the ABI 7500 Fast Real-Time PCR System (Applied Biosystems). The thermal cycling profile for both amplicons began with a 95°C incubation for 10 minutes to activate the AmpliTaq Gold DNA polymerase. For telomere PCR, this was followed by 32 cycles of 95°C for 30 seconds, 58°C for 60 seconds and 72°C for 60 seconds. For hbg PCR, the incubation step was followed by 39 cycles of 95°C for 30 seconds, 55°C for 60 seconds and 72°C for 60 seconds. The delta Cq (change in the quantification cycle value) was determined for each clinical DNA sample using the equation ΔCq = Cq (tel) - Cq (hbg). Inter- and intra-plate controls and reference DNA were also measured. The delta delta Cq was then calculated using the equation ΔΔCq = ΔCq (participant DNA) – ΔCq (reference DNA). The relative telomere length is the T/S ratio, where T is the amount of telomere (T = 2-Ct telomere) and S is the amount of the single copy gene (S = 2-Ct hbg): T/S = 2-Ct telomere/2-Ct HBG = 2-(Ct telomere-Ct HBG) = 2-ΔC.

### Study integrity

Several ethical considerations need to be addressed before data collection. These included the collection of data on events or experiences that could be stressful or unpleasant; protecting the safety of women who identify affirmatively to having been the victims of intimate partner violence, and; the collection and storage of human DNA. To manage these considerations “layered consent” was permitted. This will enable participants with the option to consent to some parts of a protocol and not others. For example, women are able to complete the questionnaire but not provide biological samples, or women can return buccal cells and completed the questionnaire without also providing serological samples.

Research into intimate relationship conflict and aggression involves by a number of methodological and ethical issues. These include inconsistency of measurement, under-reporting, confidentiality, informed consent and training of research staff [12,64]. To ensure the safety and comfort of the research participants, the project will be carried out according to the National Statement on Ethical Conduct in Human Research (2007) produced by the National Health and Medical Research Council (NHMRC) of Australia and also the World Health Organization’s Guidelines for Researching Violence against Women (2005). Moreover, the research team strictly adhered to informed consent procedures, ensuring that participants understand the purpose of the research, acknowledging the sensitive nature of questions and ensuring that participants were aware of support services should they be required (through QUT counseling service). Voluntary participation was also emphasized and participants were informed that they could participate in all or some aspects of the research or withdraw from the study at any time.

Another ethical issue is associated with the collection and storage of human genetic material. There has been considerable debate surrounding predictive genetic testing and whether the individual and their family should be informed of any mutations that may increase the risk of illness or late onset disorder [65,66]. Participants in this study were informed of the speculative nature of variations in genomic stress biomarkers and that the data would not directly benefit them. Furthermore, women were provided information on the genomic biomarker being examined and the storage of genetic material at the completion of the study.

To protect the confidentiality of participants, all questionnaires, buccal swabs, pathology forms and serology tubes will remain unnamed, with only coded numbers appearing on data. De-identified data and consent forms will be kept separately in two locked cabinets which will only be accessible by authorized research staff. Furthermore, electronic data will be kept is a secure, password protected file on the university server and will only be accessible to the research team. Finally, ethical approval will be sought from relevant human research ethics committees (HREC) before commencing this study.

### Data analysis

All statistical data will be analysed using SPSS version 19.0 and AMOS version 19.0 statistical packages [67]. The demographic characteristics of the sample will be reported as means, and standard deviations for continuous variables, and frequencies and percentages for categorical variables. Descriptive exploration of the main independent variable will determine the frequency of occurrence, percentage, and rank order of each type of SLEs; the total number of SLEs exposures will then be calculated by summing the number of affirmative responses given on the LSC-R for each participant. Linear mixed models (LMM) will be used to address the main research questions. LMM enable the researcher to examine both differences in subject-level predictor variables (e.g., extent and chronicity of SLEs) and random between-subject variance in trajectories (HRQoL) in a given longitudinal data set and to assess how much variance between subjects remains after including fixed effects in a model [68]. Furthermore, LMM has demonstrated effectiveness in genome-wide association studies whereby carefully selecting a small number of DNA sequences may reduce the risk of error and bias, increase confidence in findings, and reduce computational cost [69].

## Discussion

Australia’s investment in preventative healthcare and healthy aging are high priorities. Although women’s life expectancy in Australia and many other industrialized countries is generally good, there are some notable health inequalities associated with life events [3,5,7,20,70], social and environmental factors [1,20] and unhealthy lifestyle [36]. Continued improved health depends on further exploring the factors associated with morbidity and mortality in women particularly as aging may pose a cumulative risk of exposure to stress. It is through the identification of the factors contributing to poor health that we are able to develop socially, medically and ethical responsive strategies to meet women’s health needs thereby helping women to live healthy, productive and fulfilling lives. This study draws on a research team from social and biomedical sciences with extensive and complementary skills in research and praxis regarding women’s health, stress, quality of life and clinical biomarkers. The aim of the research is to further our understanding of the processes associated with prolonged exposure to stress and will advance knowledge and understanding of the relationships between stress, quality of life, stress biomarkers, bodily function and cell senescence [22-25]. It is hoped that by identifying the factors which may contribute to poor health in women, we will improve the evidence base for recommendations for women’s health thus potentially reducing future chronic health problems.

### Methodological considerations

The potential impact of attrition over time must be considered. Like many other longitudinal studies in aging populations, a proportion of participants have dropped out over time and this may have has impacted on the validity of results [71]. According to Suneeta and colleagues, attrition is more common amongst older, fail, cognitively impaired, less educated or poor functioning participants [72]. The authors found participants also frequently report a variety of practical reasons for withdrawal including the study being too time-consuming and problems with transport [71,72]. Indeed, for women in this study, travelling to a pathology collection centre for fasting blood samples may have presented a burden for some women.

To assess attrition, the socio-demographic profile of the sample was compared to similarly aged Queensland women. Comparisons between Queensland women aged 55-74 years using census data [73] and the 2011 HOW sample, showed no statistically differences in terms of marital status, educational attainment, Aboriginal or Torres Strait Islander ancestry, country of birth, or employment status. Detailed analysis of the HOW sample was performed by comparing the retained sample with women who were lost to follow-up. Data from 2006 and 2011 was examined across a range of variables (age, identifying as Aboriginal or Torres Strait Islander, country of birth, highest educational attainment, employment status and income) to determine whether those who were lost to follow-up were systematically different from women who remained in the study. Overall, women who were lost to follow-up were less likely to report being retired (19% versus 29%) and more likely to report being permanently ill or unable to work (8% versus 2%) than women who continued in the study (χ2 = 15.3, df = 5, p = 0.009). This is consistent with Goldberg and colleagues who also found that reported that retirement was negatively associated with attrition [72,74].

## Abbreviations

HRQoL: Health-related quality of life; HOW: Healthy Aging of Women study; SLE: Stressful life event; CVD: Cardiovascular disease; HPA: Hypothalamic-pituitary-adrenal; HTN: Hypertension; SNS: Sympathetic nervous system; STD: Sexually transmitted disease; DNA: Deoxyribonucleic acid; LSC-R: Life Stressor Checklist – Revised; CTS2: Revised Conflict Tactics Scale; PCR: Polymerase chain reaction; HREC: Human research ethics committee; LMM: Linear mixed models.

## Competing interests

No competing financial interests exist.

## Authors’ contributions

CS, EW, JH, KL, LC, DA conceived of the study, and participated in its design and coordination and helped to draft the manuscript. CS, EW, PT, GC, AS, LC, DA developed the protocol for the collection and processing of buccal cell and blood samples. EW, PT, GC, AS optimized and performed the relative telomere length PCR assay. EW, AO, MK carried out the carried out the immunoassays. CS, EW, LC, DA drafted the manuscript. CS, EW, GC performed the statistical analysis. All authors read and approved the final manuscript.

## Authors’ information

Debra Anderson: Senior author

## Pre-publication history

The pre-publication history for this paper can be accessed here:

http://www.biomedcentral.com/1471-2458/14/9/prepub
